# Association of Maternal Dietary Habits and Infant *MTHFR* Gene Polymorphisms with Ventricular Septal Defect in Offspring: A Case–Control Study

**DOI:** 10.3390/nu16132005

**Published:** 2024-06-24

**Authors:** Xiaorui Ruan, Ziye Li, Taowei Zhong, Ridan Lei, Manjun Luo, Mengting Sun, Jiabi Qin

**Affiliations:** Department of Epidemiology and Health Statistics, Xiangya School of Public Health, Central South University, Changsha 410013, China; xiaoruiruan01@gmail.com (X.R.); liziye_ah@163.com (Z.L.); taoweizhong98@163.com (T.Z.); lmj1990dlh@163.com (M.L.);

**Keywords:** ventricular septal defect, *MTHFR* gene, diet, gene–environment interactions

## Abstract

This study aimed to explore the association of maternal diet, infant *MTHFR* gene polymorphisms, and their interactions with the risk of ventricular septal defects (VSDs). This case–control study recruited 448 mothers of VSD children and 620 mothers of healthy counterparts. Multivariable-adjusted logistic regression models were constructed to examine the association between maternal dietary habits during the first trimester of gestation, *MTHFR* gene polymorphisms, and VSD. Gene–environment interaction effects were analyzed through logistic regression models, with false discovery rate *p*-value (FDR_*p*) < 0.05. Maternal excessive intake of fermented bean curd (OR = 2.00, 95%CI: 1.59–2.52), corned foods (OR = 2.23, 1.76–2.84), fumatory foods (OR = 1.75, 1.37–2.23), grilled foods (OR = 1.34, 1.04–1.72), and fried foods (OR = 1.80, 1.42–2.27) was associated with an increased risk of VSD. Regular intake of fish and shrimp (OR = 0.42, 0.33–0.53), fresh eggs (OR = 0.58, 0.44–0.75), soy products (OR = 0.69, 0.56–0.85), and dairy products (OR = 0.71, 0.59–0.85) was found to reduce the occurrence of VSD. Moreover, *MTHFR* gene polymorphisms at rs2066470 (homozygous: OR = 4.28, 1.68–10.90), rs1801133 (homozygous: OR = 2.28, 1.39–3.74), and rs1801131 (heterozygous: OR = 1.75, 1.24–2.47; homozygous: OR = 3.45, 1.50–7.95) elevated offspring susceptibility to VSDs. Furthermore, significant interactions of *MTHFR* polymorphisms with maternal dietary habits were observed, encompassing corned foods, fermented bean curd, fried foods, and grilled foods. Maternal dietary habits; *MTHFR* polymorphisms at rs2066470, rs1801131, and rs1801133; and their interactions were significantly associated with the occurrence of VSDs in offspring.

## 1. Introduction

Congenital heart disease (CHD) is recognized as the most prevalent congenital malformation worldwide, presenting in approximately 1% of newborns and causing 217,000 deaths in 2019 globally [1,2]. In China, ventricular septal defects (VSDs) occur as the most frequent subtype of CHD, with an estimated prevalence of 1.410 per 1000 births [3]. The reported average inpatient costs range from USD 3275 to 9409 [4,5], demonstrating that CHD (including VSDs) remains a critical public health concern. It is commonly understood that genetic and environmental factors are involved in the pathogenesis of most non-syndromic CHDs. Despite extensive etiological investigations, merely ~15% of the origin of CHD has been disclosed [6]. Currently, about 400 genes have been discovered to be related to CHD, including those encoding transcription factors, cell signaling molecules, and structural proteins participating in heart development [7]. Epigenetic alternations, particularly via promoter hypermethylation, have been identified as contributors as well [8,9]. Collectively, single-nucleotide polymorphisms (SNPs) resulting in deleterious missense variations play a crucial role in understanding genotype–phenotype correlations. Of note, 8% of CHD etiology can be attributed to de novo autosomal-dominant variants [10]. 

Previous studies have pointed out that polymorphisms of genes implicated in the folate–homocysteine pathway were associated with the occurrence of CHD, encompassing methylenetetrahydrofolate reductase (*MTHFR*) [11,12], cysteine acetylase (*CBS*) [13], methionine synthase (*MTR*) [14], and methionine synthase reductase (*MTRR*) [15]. The prevailing *MTHFR* gene encodes the essential enzyme involved in the one-carbon pathway, converting 5,10-methylenetetrahydrofolate to 5-methyltetrahydrofolate. This process is catalyzed by *MTR*, with cofactor vitamin B_12_, when a methyl donor remethylates homocysteine to methionine [16]. Polymorphisms of the *MTHFR* gene result in impaired enzyme activity and disturbed clearance of homocysteine and subsequently present with hyperhomocysteinemia, the acknowledged teratogen and risk factor of CHD, presumably due to oxidative stress [17,18,19]. Epidemiological evidence has documented associations between maternal and infant *MTHFR* gene polymorphisms at rs1801133 and rs1801131 and CHD [12,14,20]. The current study focuses on the relationship between offspring *MTHFR* gene polymorphisms and VSDs, the major subtype of CHD, and this investigation extends beyond the two loci mentioned above.

In addition to the genetic basis of CHD, environmental factors (including lifestyle factors) contribute to 2% of pathogenesis [21], suggesting a determining effect of gene–environment interactions. As a modifiable lifestyle factor, dietary habits could be subjected to an intervention in a cost-effective and low-risk manner. It has been proved that maternal folic acid (vitamin B_9_) supplementation before and during early gestation decreased susceptibility to CHD, including VSDs, in offspring [22,23]. Moreover, our research group has expounded on the interaction effects between maternal folic acid use and folate receptor gene polymorphisms on VSD occurrence [24]. Similarly, retinoic acid (derived from dietary vitamin A) was found to interact with the retinaldehyde dehydrogenase 2 (*Raldh2*) gene in the formation of the murine outflow tract [25]. These studies highlight the value of probing into the interactions of maternal dietary intake and SNPs of target genes. Considering dietary factors vary with disparate regions, ethnicities, and socioeconomic statuses, a general evaluation of dietary habits rather than an evaluation of a single nutrient or food species would provide all-inclusive information. 

Therefore, we conducted this case–control study to explore the association of infant *MTHFR* genetic polymorphisms, maternal dietary habits, and their interactive effects with the susceptibility to VSDs in offspring. 

## 2. Materials and Methods

### 2.1. Study Design and Participants

The present study was designed as a case–control study conducted from November 2018 to January 2021 in Changsha City, Hunan Province, China. Children diagnosed with VSD through Doppler echocardiography and confirmed by surgery were assigned to the case group, along with their mothers. During the same period, children without any congenital malformations after health consultation or physical examination were assigned to the control group, along with their mothers. The case and control groups were recruited from the Department of Cardiothoracic Surgery and the Department of Child Health of Hunan Provincial Children’s Hospital, respectively. VSD children were consecutively recruited, while controls were randomly selected. All children included in the study were under 1 year old to minimize recall bias regarding maternal exposure before pregnancy to the first trimester. The inclusion criteria required Chinese Han descent, no artificial fertilization, completion of a questionnaire, spontaneous pregnancy, informed consent, and provision of a blood sample. Additionally, syndromic cases with extra-cardiovascular abnormalities or chromosomal malformations were excluded from this research. This study was performed following the principles of the Declaration of Helsinki. 

### 2.2. Variables

In the present study, the primary outcome of interest was non-syndromic VSD. As previously outlined, these VSD cases were initially diagnosed utilizing Doppler echocardiography and subsequently confirmed via surgery. The study focused on two main exposures of interest—SNPs of the infant *MTHFR* gene and maternal dietary habits during the first trimester, which were assessed through a self-administered questionnaire. The questionnaire was designed on the basis of previous research, Chinese dietary guidelines, and local eating habits [26,27]. In addition, a pre-survey of the questionnaire displayed satisfactory internal consistency (Cronbach’s alpha = 0.806) and test-retest reliability (intraclass correlation coefficient = 0.852). This questionnaire was comprised of 12 items on the dietary habits of mothers during early pregnancy, covering fermented bean curd, corned foods, fumatory foods, grilled foods, fried foods, fresh meat, fish and shrimp, fresh eggs, fresh vegetables, fresh fruit, soy products, and dairy products. Intake frequencies were categorized into hardly (i.e., ≤2 times per week), sometimes (i.e., 3–5 times per week), and often (i.e., ≥6 times per week).

Other covariates were collected based on existing literature [28,29,30,31], including sex, maternal age at pregnancy, residence, last year’s household income, maternal schooling years, pregestational BMI, history of adverse pregnancy outcomes, history of familial congenital malformations, consanguineous marriages, gestational hypertension during this pregnancy, gestational diabetes mellitus during this pregnancy, lifestyle factors during the first trimester of pregnancy (i.e., smoke and drink alcohols), pregestational folic acid supplementation, and pregestational exposure to antibiotics. A portion of the above questions were documented in the nationwide free prenatal health care system [32]. Certified inquirers conducted face-to-face interviews with mothers to complete the questionnaires.

### 2.3. Selection of MTHFR SNPs and Genotyping

Peripheral venous blood (3–5 mm) was drawn from each child to determine their genotype. Blood samples were transported to the laboratory at a low temperature (≤4 °C) within 12 h and subsequently centrifuged to separate blood cells and plasma. Until genotyping, blood cells were extracted and stored at an ultra-low temperature refrigerator (−80 °C). According to previously published literature [31], we selected candidate loci of the *MTHFR* gene. The process of selecting *MTHFR* SNPs from the HapMap database was performed by the SNPBrowserTM program (version 3.0) provided by Applied Biosystems Inc. (Beijing, China). The selection criteria included pairwise r^2^ ≥ 0.8 and minor allele frequencies (MAF) > 0.1. Ultimately, rs2274976, rs4846052, rs7525338, rs4846051, rs1476413, rs2066470, rs1801133, and rs1801131 were included as candidate loci in this study.

Genomic DNA was extracted from peripheral venous blood using the QIAamp DNA Blood Mini Kit (Qiagen, Valencia, CA, USA). The DNA was then dissolved in a sterile TBE buffer in accordance with the manufacturer’s protocol. Utilizing a matrix-assisted laser desorption and ionization time-of-flight mass spectrometry system (MassARRAY; Agena iPLEX assay, San Diego, CA, USA), genotyping was carried out. The samples were grouped in a manner that was blind to the lab technicians responsible for conducting the genotyping and recording the genotype data to reduce selection bias. To ensure the integrity of individual genotypes, we implemented a minimum 50% SNP genotyping call rate.

### 2.4. Statistical Analyses

For categorical data, the distribution of individual characteristics was presented as a number (percentage). We performed the Chi-squared test and Wilcoxon rank-sum test (for ordinal variables) to analyze differences between the case and control groups. Additionally, we conducted the Hardy-Weinberg equilibrium (HWE) test on genotype frequency in the control group, with a significant level set at 0.05.

To estimate the strength of the association between *MTHFR* gene polymorphisms, maternal dietary habits, and VSD susceptibility, we employed univariate and multivariate logistic regression models, providing unadjusted and adjusted odds ratios (ORs) with 95% confidence intervals (CIs). Furthermore, we assessed the interaction effects between *MTHFR* gene polymorphisms and maternal dietary habits using logistic regression models. All statistical analyses were conducted utilizing R software version 4.2.2. To minimize type I error induced by multiple testing, Benjamini and Hochberg’s correction was applied to each *p*-value, with a false discovery rate *p*-value (FDR_*p*) < 0.05 regarded statistically significant.

## 3. Results

### 3.1. Participants’ Baseline Characteristics

A total of 1068 eligible participants were enrolled in this study, consisting of 620 individuals diagnosed with VSD, 448 non-VSD children, and their mothers. Baseline characteristics of the case and control group are presented in Table 1, revealing notable differences between the two groups regarding sex, residence, last year’s household income, maternal schooling years, pregestational BMI, history of adverse pregnancy outcomes, history of familial congenital malformations, consanguineous marriages, gestational hypertension, gestational diabetes mellitus, smoking during the first trimester of pregnancy, alcohol consumption during the first trimester of pregnancy, and pregestational exposure to antibiotics. These variables were regarded as confounders and adjusted for in analyzing the associations between offspring *MTHFR* gene polymorphisms, maternal dietary habits, and their interactions with VSD risk.

### 3.2. Association between Maternal Dietary Factors and VSD in Offspring

**Figure 1:**
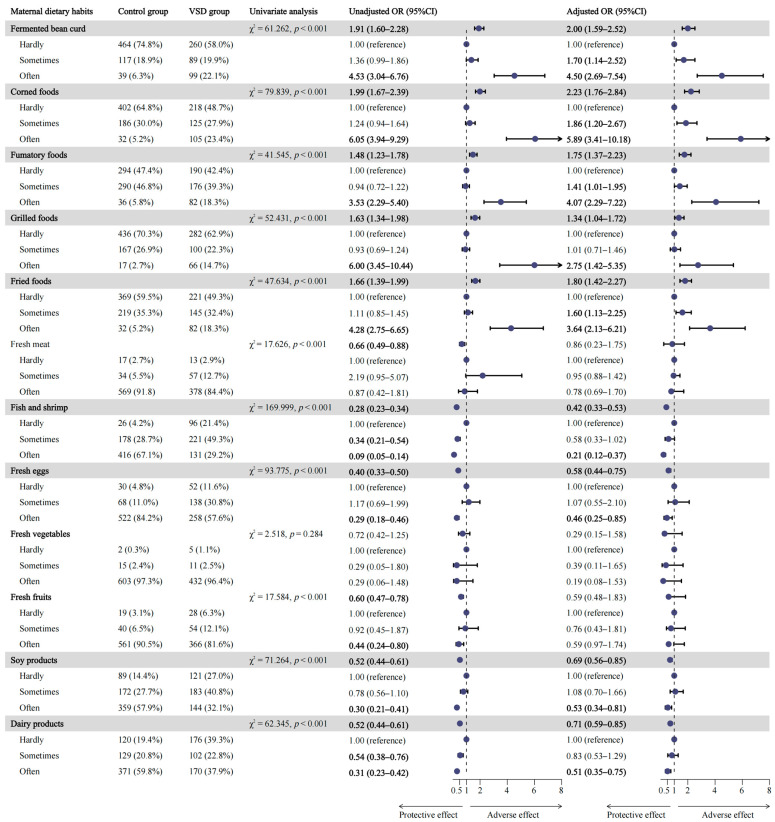
The association between maternal dietary habits and the risk of VSD in offspring. Abbreviation: VSD ventricular septal defect. Adjusted for sex, residence, last year’s household income, maternal schooling years, pregestational BMI, history of adverse pregnancy outcomes, history of familial congenital malformations, consanguineous marriages, gestational hypertension, gestational diabetes mellitus, smoking during the first trimester of pregnancy, drinking alcohol during the first trimester of pregnancy, pregestational exposure to antibiotics.

Figure 1 summarizes the frequency of maternal consumption of 12 food categories during the first trimester and their associations with VSD risk. Multivariate logistic regression analyses showed that maternal excessive intake of fermented bean curd (OR = 2.00, 95%CI: 1.59–2.52), corned foods (OR = 2.23, 95%CI: 1.76–2.84), fumatory foods (OR = 1.75, 95%CI: 1.37–2.23), grilled foods (OR = 1.34, 95%CI: 1.04–1.72), and fried foods (OR = 1.80, 95%CI: 1.42–2.27) was significantly associated with an increased risk of VSD in offspring. Conversely, consumption of fish and shrimp (OR = 0.42, 95%CI: 0.33–0.53), fresh eggs (OR = 0.58, 95%CI: 0.44–0.75), soy products (OR = 0.69, 95%CI: 0.56–0.85), and dairy products (OR = 0.71, 95%CI: 0.59–0.85) was found to be associated with a reduced VSD occurrence in offspring.

### 3.3. Association between Infant MTHFR Gene Polymorphisms and the Risk of VSD

**Figure 2:**
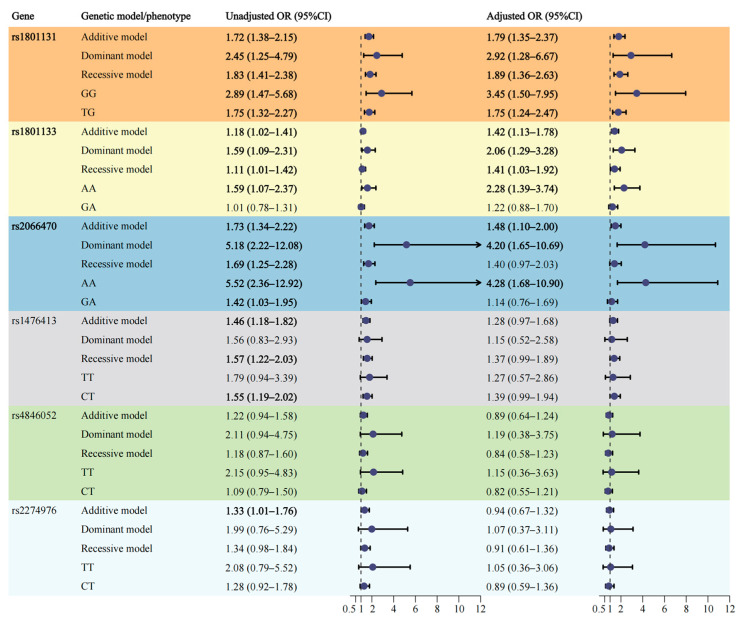
Association between *MTHFR* gene polymorphisms and risk of VSD in offspring. Abbreviation: VSD ventricular septal defect, MTHFR Methylenetetrahydrofolate reductase. Adjusted for sex, residence, last year’s household income, maternal schooling years, pregestational BMI, history of adverse pregnancy outcomes, history of familial congenital malformations, consanguineous marriages, gestational hypertension, gestational diabetes mellitus, smoking during the first trimester of pregnancy, drinking alcohol during the first trimester of pregnancy, pregestational exposure to antibiotics.

The genotype distribution of the *MTHFR* gene in the control group adhered to HWE, as indicated in Table 2. (All *p*-values > 0.05). Due to the limited numbers of variant genotypes, rs4846051 and rs7525338 were excluded from further analysis. Figure 2 provides a summary of the association of the *MTHFR* gene polymorphisms with VSD risk. Results from multivariate logistic regression analyses showed significant associations between genetic polymorphisms of the *MTHFR* gene at rs2066470 (AA vs. GG: OR = 4.28, 95%CI: 1.68–10.90; the dominant model: OR = 4.20, 95%CI: 1.65–10.69; the additive model: OR = 1.48, 95%CI: 1.10–2.00), rs1801133 (AA vs. GG: OR = 2.28, 95%CI: 1.39–3.74; the recessive model: OR = 1.41, 95%CI: 1.03–1.92; the dominant model: OR = 2.06, 95%CI: 1.29–3.28; the additive model: OR = 1.42, 95%CI: 1.13–1.78), and rs1801131 (TG vs. TT: OR = 1.75, 95%CI: 1.24–2.47; GG vs. TT: OR = 3.45, 95%CI: 1.50–7.95; the recessive model: OR = 1.89, 95%CI: 1.36–2.63; the dominant model: OR = 2.92, 95%CI: 1.28–6.67; the additive model: OR = 1.79, 95%CI: 1.35–2.37) and an increased susceptibility to VSD.

### 3.4. Interaction of Infant MTHFR Genetic Polymorphisms and Maternal Dietary Habits

Table 3 summarizes the multiplicative interaction effects of the *MTHFR* genetic polymorphisms and maternal dietary habits on VSD risk based on logistic regression analyses. Significant interactions were observed between the variant genotypes (GA + AA) of rs2066470 and maternal excessive intake of fried food (OR = 1.37, 95%CI: 1.14–1.64, FDR_*p* < 0.05). Moreover, a strong interaction effect was detected between the polymorphism of rs1801133 and maternal overconsumption of fermented bean curd (OR = 6.25, 95%CI: 2.04–19.12, FDR_*p* < 0.05). Furthermore, children carrying mutations at rs1801131 had higher odds of VSD when their mothers consumed more corned foods (OR = 1.81, 95%CI: 1.50–2.18), fermented bean curd (OR = 1.60, 95%CI: 1.39–1.83), fried foods (OR = 1.64, 95%CI: 1.39–1.95), and grilled foods (OR = 2.78, 95%CI: 1.38–5.57) (all FDR_*p* < 0.05).

## 4. Discussion

Both modifiable environmental factors and irrevocable gene polymorphisms, along with their interaction effects, are extensively acknowledged to play vital roles in the pathogenesis of CHD [10]. Considering the tangled interplay among diverse food categories, we collected food frequencies during the first trimester (the critical period of teratogenesis) rather than investigating one particular nutrient [13,33]. This study examined the correlation between maternal diet habits during early pregnancy and the risk of VSD in offspring. Our findings indicate that mothers who consumed more fermented bean curd (OR = 2.00), corned foods (OR = 2.23), fumatory foods (OR = 1.75), grilled foods (OR = 1.34), and fried foods (OR = 1.80) during early gestation significantly increased their offspring’s susceptibility to VSD.

The strongest association (OR = 5.89) was observed between maternal frequent consumption of corned foods (≥6 times per week) during the first trimester and VSD in offspring. This may be attributed to the excessive intake of sodium when consuming corned foods since high-salt-induced reactive oxygen species interfered with the migration of cardiac progenitor cells and their gene expression [34]. Our findings demonstrated the adverse impact of fermented bean curd intake on heart defects in offspring (3–5 times per week: OR = 1.70; ≥6 times per week: OR = 4.50). On the contrary, a randomized controlled trial conducted on healthy individuals revealed that consuming a meal containing fermented bean curd lowered serum homocysteine levels, albeit without statistical significance [35]. This suggests a potential protective role of fermented bean curd on cardiac, given that hyperhomocysteinemia is a known risk factor for VSD [36]. The difference is presumably due to high salt consumption accompanied by fermented bean curd [37]. Polycyclic aromatic hydrocarbons (PAHs), specifically benzo(a)pyrene (BaP), are frequently observed in fumatory foods [38]. Taking into account its ability to pass through the embryonic and fetal hemato–encephalic barrier, PAHs are suspected to be a teratogen with mutagenic effects and oxidative stress impairment [39,40]. Li and his co-workers have expounded on the positive association of maternal exposure to PHAs with the risk of CHD in offspring, including septal defects [41].

When processed using high-temperature cooking strategies such as grilling and deep-frying, ingredients rich in carbohydrates and proteins produce advanced glycated end products (AGEs) through the Maillard reaction [42]. AGEs are widely documented to be associated with various disorders, encompassing diabetes mellitus (notably, gestational diabetes), impaired fertility, such as polycystic ovary syndrome (PCOS), renal diseases, and gastrointestinal dysfunction [43,44]. Significantly, it has been revealed that elevated AGEs increase the risk of cardiovascular events by damaging artery endothelial and cardiac muscle cells [45]. In addition, researchers have reported that a higher level of the soluble receptors for AGEs, an indicator of AGEs level, caused embryonic lethality mainly due to oxidative stress, inflammation, and thrombotic reactions in uteroplacental tissue as well as limited uterine blood flow [46]. This evidence supports our finding that additional consumption of grilled and fried foods during early pregnancy, which increases the intake of exogenous dietary AGEs, could be harmful to human embryos. Apart from AGEs, undesirable byproducts of thermal processing include acrylamide and heterocyclic aromatic amines, which are putative sources of cardiovascular toxicity, reproductive toxicity, teratogen, carcinogenicity, and neurotoxicity [47,48]. In contrast to the harmful effects of unhealthy foods, we identified the probable protective dietary factors of VSD, including fish and shrimp (OR = 0.42), fresh eggs (OR = 0.58), soy products (OR = 0.69), and dairy products (OR = 0.71), consistent with our previous publication [13].

Folate acid-homocysteine metabolic pathway is implicated in the formation of cardiovascular disease, CHD, and other birth defects [20,21,22]. Regarding the extensively studied *MTHFR* gene in the one-carbon cycle, two loci have undergone in-depth exploration: C677T (rs1801133) and A1298C (rs1801131). The *MTHFR* rs1801133 polymorphism involves a C-to-T substitution, leading to an Ala-to-Val change at base pair 677, while rs1801131 polymorphism causes a Glu–Ala change due to an A-to-C substitution. Both alterations are associated with reduced enzyme activity [18,49]. Research concerning these two loci has yielded inconsistent findings. Wang et al. found that both infant (pooled OR = 1.30) and maternal (pooled OR = 1.16) *MTHFR* rs1801133 polymorphisms were associated with increased susceptibility to CHD [20]. Similarly, Shi et al. identified maternal *MTHFR* gene mutation at rs1801131 as a marker for CHD (heterozygous: OR = 2.04; dominant model: OR = 1.89) [14]. Furthermore, two studies among the Pakistani population provided positive associations of the VSD subtype [50,51]. However, Lee and colleagues comprehensively analyzed 11 meta-analyses regarding the relationship between *MTHFR* gene polymorphisms at rs1801133 and CHD and revealed that four studies did not support their associations [52].

Our research team has priorly demonstrated the associations of maternal *MTHFR* rs1801131 polymorphisms with CHD (OR = 5.18) and VSD subtype (OR = 4.98), while no such correlation was observed for rs1801133 with target diseases [11]. Moreover, our published article indicated that infant *MTHFR* gene polymorphisms increased the risk of CHD (rs2066470: OR = 5.09; rs1801133: OR = 2.49; rs1801131: OR = 1.84) [12]. In the present study, we further explored the SNPs of the infant *MTHFR* gene, maternal dietary habits, and the impact of gene–environment interactions on VSD, the predominant phenotype of cardiac defects. Likewise, rs2066470 (homozygous: OR = 4.28), rs1801133 (homozygous: OR = 2.28), and rs1801131 (heterozygous: OR = 1.75; homozygous: OR = 3.45) polymorphisms in pediatric *MTHFR* gene were observed to be associated with VSD. Building on our previous discovery that the interaction between *MTHFR* gene polymorphisms and lack of maternal folic acid supplementation elevated CHD susceptibility, this research documented significant multiplicative interactions between offspring *MTHFR* gene polymorphisms and maternal dietary habits during the first trimester. Specifically, maternal additional consumption of corned foods, fermented bean curd, fried foods, and grilled foods raised the odds of VSD when their offspring carried mutations in the *MTHFR* gene. These findings were revealed for the first time. Mouse models disclosed that the *MTHFR* deficiency interacted with dietary riboflavin in embryonic heart development. Nonetheless, the exact mechanisms underlying our novel observations remain unclear and more research is requisite. Furthermore, future research is warranted to investigate the interactions between maternal *MTHFR* gene polymorphisms and gestational diet, enhancing the understanding of complex interplay between feeding pattern and genetic factors.

There are still some limitations of our study that should be considered. First, we designed a hospital-based study and recruited participants from Hunan Children’s Hospital. Although we have tried to refine the study design and execute it rigorously, our findings need further validation through cohort studies or prospective research. Second, recall bias was unavoidable when collecting maternal dietary habits during early gestation, despite our multiple efforts to mitigate it. We attempted to address this by limiting the age of infants to one year. Thirdly, unmeasured confounders could potentially influence the outcomes and introduce bias into our results. Finally, given the ethnic and regional differences in genetic polymorphisms, the results should be interpreted with limited extrapolation.

## 5. Conclusions

In this hospital-based case–control study, polymorphisms of the infant *MTHFR* gene at rs2066470, rs1801133, and rs1801131 were found to be associated with VSD in offspring. Moreover, we observed the associations between VSD and maternal dietary habits during early gestation. Notably, we identified interactions between infant *MTHFR* gene polymorphisms and maternal overconsumption of corned foods, fermented bean curd, fried foods, and grilled foods. These findings were expected to stratify gestational individuals based on their genotypes and implement dietary recommendations accordingly.

## Figures and Tables

**Table 1:** Demographic characteristics among 448 VSD cases and 620 healthy controls.

Variables	Control Group (%)	VSD Group (%)	Univariable Analysis
Sex			*χ*^2^ = 13.839, *p* < 0.001
Female	213 (34.4)	205 (45.8)	
Male	407 (65.6)	243 (54.2)	
Maternal age at pregnancy			*Z* = 0.734, *p* = 0.654
<25	124 (20)	110 (24.6)	
25–29	261 (42.1)	183 (40.8)	
30–34	152 (24.5)	94 (21)	
≥35	83 (13.4)	61 (13.6)	
Residence			*χ*^2^ = 22.977, *p* < 0.001
Rural	342 (55.2)	312 (69.6)	
Urban	278 (44.8)	136 (30.4)	
Last year’s household income (RMB)			*Z* = 8.051, *p* < 0.001
≤50,000	179 (28.9)	353 (78.8)	
60,000–100,000	267 (43.1)	66 (14.7)	
110,000–150,000	57 (9.2)	9 (2)	
≥160,000	117 (18.9)	20 (45)	
Maternal schooling years			*Z* = 5.774, *p* < 0.001
<9	7 (1.1)	43 (9.6)	
9–12	117 (18.9)	207 (46.2)	
13–16	217 (35)	122 (27.2)	
≥17	279 (45)	76 (17)	
Pregestational BMI (kg/m^2^)			*χ*^2^ = 15.064, *p* = 0.005
<18.5	157 (25.3)	82 (18.3)	
18.5–23.9	343 (55.3)	292 (65.2)	
24.0–27.9	78 (12.6)	46 (10.3)	
≥28.0	42 (6.8)	28 (6.2)	
History of adverse pregnancy outcomes			*χ*^2^ = 18.775, *p* < 0.001
No	349 (56.3)	192 (42.9)	
Yes	271 (43.7)	256 (57.1)	
History of familial congenital malformations			*χ*^2^ = 12.494, *p* < 0.001
No	618 (99.7)	435 (97.1)	
Yes	2 (0.3)	13 (2.9)	
Consanguineous marriages			*χ*^2^ = 11.582, *p* = 0.001
No	617 (99.5)	434 (96.9)	
Yes	3 (0.5)	14 (3.1)	
Gestational hypertension			*χ*^2^ = 29.240, *p* < 0.001
No	611 (98.5)	411 (91.7)	
Yes	9 (1.5)	37 (8.3)	
Gestational diabetes mellitus			*χ*^2^ = 26.537, *p* < 0.001
No	603 (97.3)	402 (89.7)	
Yes	17 (2.7)	46 (10.3)	
Smoking during the first trimester of pregnancy			*χ*^2^ = 12.190, *p* < 0.001
No	614 (99)	429 (95.8)	
Yes	6 (1)	19 (4.2)	
Drinking alcohol during the first trimester of pregnancy			*χ*^2^ = 12.843, *p* < 0.001
No	598 (96.5)	409 (91.3)	
Yes	22 (3.5)	39 (8.7)	
Pregestational folic acid supplementation			*χ*^2^ = 3.325, *p =* 0.068
No	43 (6.9)	45 (10)	
Yes	577 (93.1)	403 (90)	
Pregestational exposure to antibiotics			*χ*^2^ = 17.135, *p* < 0.001
No	601 (96.9)	408 (91.1)	
Yes	19 (3.1)	40 (8.9)	

Abbreviation: BMI, body mass index.

**Table 2:** Genotype frequencies and HWE test of *MTHFR* gene loci among 448 VSD cases and 620 healthy controls.

SNPs	Major Allele	Minor Allele	Chromosome	Group	Genotype Frequency n (%)			HWE Test *p*
					AA	AB	BB	
rs2274976	C	T	Chr1:11790870	Control	522 (84.2)	91 (14.7)	7 (1.1)	0.1856
				Case	358 (79.9)	80 (17.9)	10 (2.2)	
rs4846052	C	T	Chr1:11797894	Control	505 (81.5)	105 (16.9)	10 (1.6)	0.1012
				Case	353 (78.8)	80 (17.9)	15 (3.3)	
rs1476413	C	T	Chr1:11792243	Control	437 (70.5)	164 (26.5)	19 (3.1)	0.4532
				Case	270 (60.3)	157 (35.0)	21 (4.7)	
rs2066470	G	A	Chr1:11803000	Control	515 (83.1)	98 (15.8)	7 (1.1)	0.3425
				Case	333 (74.3)	90 (20.1)	25 (5.6)	
rs1801133	G	A	Chr1:11796321	Control	286 (46.1)	275 (44.4)	59 (9.5)	0.5431
				Case	195 (43.5)	189 (42.2)	64 (14.3)	
rs1801131	T	G	Chr1:11794419	Control	457 (73.7)	149 (24.0)	14 (2.3)	0.6536
				Case	271 (60.5)	153 (34.2)	24 (5.4)	

Abbreviation: HWE Hardy-Weinberg equilibrium, SNPs single-nucleotide polymorphisms. AA homozygous with minor allele, AB heterozygous, BB homozygous with major allele.

**Table 3:** Interactions of infant *MTHFR* gene polymorphisms and maternal dietary habits based on multivariate logistic regression among 448 VSD cases and 620 healthy controls.

Dietary Habits	rs2066470		rs1801133		rs1801131	
	aOR (95%CI)	FDR_*p*	aOR (95%CI)	FDR_*p*	aOR (95%CI)	FDR_*p*
Corned foods	0.74 (0.33–1.66)	0.661	3.10 (1.08–8.89)	0.116	1.81 (1.50–2.18)	<0.001
Fermented bean curd	0.89 (0.41–1.94)	0.777	6.25 (2.04–19.12)	0.010	1.60 (1.39–1.83)	<0.001
Fried foods	1.37 (1.14–1.64)	0.005	1.20 (0.43–3.36)	0.801	1.64 (1.39–1.95)	<0.001
Fumatory foods	0.65 (0.25–1.71)	0.650	2.26 (1.21–4.25)	0.055	1.02 (0.57–2.13)	0.859
Fresh meat	1.62 (0.72–3.62)	0.242	2.28 (1.06–4.93)	0.061	0.65 (0.31–1.37)	0.257
Fresh fruit	0.67 (0.57–3.62)	0.197	0.56 (0.29–1.11)	0.095	0.51 (0.34–2.52)	0.614
Grilled foods	1.23 (1.03–1.49)	0.083	0.60 (0.31–1.17)	0.222	2.78 (1.38–5.57)	0.010
Fish and shrimp	0.98 (0.84–1.02)	0.275	1.05 (0.91–1.21)	0.631	0.72 (0.22–2.33)	0.724
Fresh eggs	1.09 (0.92–1.30)	0.636	1.13 (0.97–1.30)	0.111	0.48 (0.08–2.98)	0.723
Fresh vegetables	0.82 (0.71–1.03)	0.653	1.11 (0.94–1.37)	0.143	0.89 (0.75–1.56)	0.999
Soy products	0.97 (0.88–1.08)	0.675	1.10 (0.96–1.26)	0.251	1.55 (0.48–4.97)	0.666
Dairy products	0.97 (0.88–1.07)	0.676	0.96 (0.86–1.15)	0.960	0.46 (0.17–1.28)	0.278

Abbreviation: aOR adjusted odds ratio, CI confidence interval. Adjusted for sex, residence, last year’s household income, maternal schooling years, pregestational BMI, history of adverse pregnancy outcomes, history of familial congenital malformations, consanguineous marriages, gestational hypertension, gestational diabetes mellitus, smoking during the first trimester of pregnancy, drinking alcohol during the first trimester of pregnancy, pregestational exposure to antibiotics.

## Data Availability

Data are unavailable due to privacy or ethical restrictions.
